# Determinants of the Uptake and Frequency of Use of a Web Portal Digital Health Intervention in Patients With Type 2 Diabetes and/or Coronary Heart Disease: Secondary Analysis of a Randomized Controlled Trial

**DOI:** 10.2196/80895

**Published:** 2026-03-25

**Authors:** Maximilian Scholl, Claas Lendt, Sebastian Appelbaum, Bianca Biallas, Katja Brenk-Franz, Chloé Chermette, Friederike Frank, Angeli Gawlik, Lisa Giesen, Martina Heßbrügge, Lucas Küppers, Larisa Pilic, Marcus Redaèlli, Lara Schneider, Frank Vitinius, Stefan Wilm, Uwe Konerding

**Affiliations:** 1 Institute of Movement Therapy and Movement-oriented Prevention and Rehabilitation, Section I, German Sports University Cologne, Cologne, Germany; 2 Department of Epidemiology and Preventive Medicine, University of Regensburg, Regensburg, Germany; 3 Department of Psychology and Psychotherapy, Witten/Herdecke University, Witten, Germany; 4 Trimberg Research Academy, University of Bamberg, An der Weberei 5, Bamberg, 96047, Germany, +49 9512973078; 5 Institute of Psychosocial Medicine, Psychotherapy and Psychooncology, Jena University Hospital, Jena, Germany; 6 Institute of Psychology, Section I: Health and Social Psychology, German Sport University Cologne, Cologne, Germany; 7 Institute for Digitalization and General Medicine, Medical Faculty, RWTH Aachen University, Aachen, Germany; 8 Center for Rare Diseases Aachen (ZSEA), Medical Faculty, RWTH Aachen University, Aachen, Germany; 9 Institute of Health Economics and Clinical Epidemiology, Faculty of Medicine and University Hospital Cologne, University of Cologne, Cologne, Germany; 10 Institute of General Practice, University of Duisburg-Essen, Essen, Germany; 11 Institute of Family Medicine and General Practice, University of Bonn, Bonn, Germany; 12 Institute of General Practice, Faculty of Medicine and University Hospital Cologne, University of Cologne, Cologne, Germany; 13 Department of Child and Adolescent Psychiatry, Psychosomatic Medicine and Psychotherapy, Faculty of Medicine and University Hospital Cologne, University of Cologne, Cologne, Germany; 14 Department of Psychosomatics and Psychotherapy, Faculty of Medicine and University Hospital Cologne, University of Cologne, Cologne, Germany; 15 Department of Psychosomatic Medicine, Robert Bosch Hospital, Stuttgart, Germany; 16 Institute of General Practice, Centre for Health and Society, Medical Faculty and University Hospital, Heinrich Heine University Düsseldorf, Düsseldorf, Germany

**Keywords:** digital health interventions, eHealth, type 2 diabetes, coronary heart disease, predictors of use, chronic disease self-management

## Abstract

**Background:**

The targeted application and design of digital health interventions (DHIs) require an understanding of usage determinants. Usage includes uptake (initial use) and frequency (extent of use), but it is unclear whether both components are driven by the same determinants.

**Objective:**

This study aimed to examine the determinants of uptake and frequency of use and assess whether they differ.

**Methods:**

The investigated DHI was a web portal provided in an intervention for improving disease-related self-management. This study is a secondary analysis of intervention group data from a parallel-group randomized controlled trial. Eligibility criteria were being an adult and being diagnosed with type 2 diabetes and/or coronary heart disease. Sociodemographic, psychological, and health-related variables were examined as determinants. Determinants were analyzed using simple and multiple regression models. Uptake was analyzed using logistic regression, and frequency was analyzed using negative binomial regression with robust SEs. Frequency was analyzed for those who used the DHI at least once. Except for sociodemographic variables, all other variables were standardized to a range from 0 to 1. For simple regression, inflation of the α error due to multiple testing was controlled via the approach of Benjamini and Hochberg, and for multiple regression, it was controlled via the significance of the complete multiple regression model.

**Results:**

Of 462 intervention group members, 199 (43.1%) used the web portal at least once. After controlling for inflation of the α error, simple regression for uptake yielded significant effects for higher education (B=0.56, 95% CI 0.18-0.95; *P*=.004), openness (B=1.08, 95% CI 0.33-1.83; *P*=.005), intention regarding physical activity (B=2.28, 95% CI 1.30-3.26; *P*<.001), and intention regarding healthy nutrition (B=2.30, 95% CI 1.30-3.31; *P*<.001). The multiple regression model for uptake was highly significant (*P*<.001), with significant positive associations for intentions regarding physical activity (B=1.86, 95% CI 0.74-2.97; *P*=.001) and healthy nutrition (B=2.22, 95% CI 1.00-3.44; *P*<.001), as well as a significant negative association for patient activation (B=−3.20, 95% CI −4.95 to −1.46; *P*<.001). After controlling for inflation of the α error, simple regression for frequency yielded no statistically significant effect, and the multiple regression model for frequency was not significant (*P*=.07).

**Conclusions:**

This study is innovative in jointly examining determinants of the uptake and frequency of use of the same DHI within a single context and sample. By demonstrating that factors driving uptake do not necessarily increase the frequency of use, it advances existing research. The study contributes to a more differentiated understanding of DHI use and shows that distinct strategies are required to promote adoption versus sustained engagement. Applying this approach to other DHIs and settings may support more targeted and equitable digital health implementation in real-world contexts, thereby optimizing digital health deployment strategies overall.

## Introduction

Chronic noncommunicable diseases, such as type 2 diabetes mellitus (T2DM) and coronary heart disease (CHD), place an enormous burden on patients and health care providers worldwide [1,2]. Improving patient self-management could help to reduce this burden [3,4]. The use of digital health interventions (DHIs), that is, interventions meant to influence health via digital tools, is a promising approach that might serve this purpose. Some examples are patient portals [5], mobile health apps [6], and web-based interventions [7]. DHIs make it possible to provide personalized, location-independent, and cost-effective support to patients [8,9]. Accordingly, DHIs have already been used in rehabilitation [10], medication adherence [11], and chronic disease management [12-15], as well as in the promotion of healthy lifestyles [16,17]. Recent systematic reviews and meta-analyses have indicated that DHIs help to improve hemoglobin A_1c_ levels [13,18,19], CHD status [14], and health-promoting behaviors, such as physical activity and healthy nutrition intake [14,20].

DHIs can only be effective if they are actually used. In some cases, repeated use might be necessary to have an impact [21,22]. Accordingly, purposeful design of interventions requires knowledge regarding the determinants of usage, that is, knowledge regarding conditions under which the DHI in question is used more and knowledge regarding the conditions under which the DHI is used less. Knowledge regarding the conditions under which the DHI is used less can be helpful in two regards: (1) it might show how use could be increased by adapting the DHI itself and/or its mode of presentation, and (2) it might show where usage is limited, that is, when neither the condition can be changed nor the DHI can be adapted. In this case, alternative interventions for the purpose addressed by the DHI in question are needed, and these might very well be nondigital interventions.

Previous research regarding potential determinants of DHI usage primarily focused on sociodemographic factors, especially age, gender, and education. Findings regarding age [23-26] and gender [24-27] were inconsistent across studies, whereas education showed a more consistent positive association with DHI usage [28-30]. Further, previous research regarding the potential determinants of DHI referred to psychological variables, such as the “big five” [29]; loneliness, depression, and anxiety [31]; health literacy [31,32]; patient activation [33,34]; and health-related behavioral intentions [26,35]. Among the “big five,” openness appeared to be positively associated with usage, whereas extraversion appeared to be negatively associated [29]. There were mixed results for mental health factors [31,35] and behavioral intentions [26,35], whereas health literacy appeared to be positively associated with usage [31,32]. Moreover, mixed results were reported for patient activation, with some studies reporting no association [36] and others reporting a positive association [37].

A major problem in research regarding the determinants of DHI usage is the operationalization of usage itself [38]. In previous research, the following two different operationalizations were mainly applied: (1) uptake, that is, the act of using the DHI at least once [26,30,39], and (2) frequency, that is, the amount of use thereafter [23-25]. Uptake should, at best, be investigated in all persons who have used the DHI, and frequency should be investigated only in those who have used it at least once. Both operationalizations constitute different components of usage. These 2 components might be determined by completely different factors. Therefore, in research regarding the determinants of DHI use, both components should be distinguished from each other. This is not only important for the sake of scientific precision but also relevant with regard to deriving implications for the targeted design of the DHI and the corresponding communication and outreach strategies. Insights into the determinants of uptake are important for the design of communication and outreach strategies, whereas insights into the determinants of frequency are relevant for the design of the DHIs themselves.

The best approach for identifying which variables affect uptake and which affect frequency is to investigate both effects for the same DHI in the same context within the same sample. There is hardly any previous research on this approach. The objective of this study is to demonstrate this approach, that is, to investigate the determinants of uptake and the frequency of use for the same DHI in the same context within the same sample.

## Methods

### Study Design

This study is a secondary analysis of a randomized controlled trial (RCT) involving a complex intervention called the Personalized Self-management Support Program (P-SUP; 2019‐2024) [40]. The P-SUP consists of the following four modules: (1) peer support groups, which met for weekly group-based exercise sessions and regularly scheduled digital expert education classes; (2) telephone coaching, which is provided to a subgroup of patients with low health literacy and low patient activation [41]; (3) feedback reports, which are provided to patients and contain routine data on medical parameters that can be used as a basis for discussing their state of health with their general practitioners during routine check-ups; and (4) a browser-based web portal, which provides practical and theoretical content on exercise, nutrition, and motivation. The last component is the DHI investigated in this study.

The P-SUP was implemented and evaluated within disease management programs (DMPs) [42] for T2DM and CHD in North Rhine-Westphalia, Germany. DMPs are structured treatment programs that address individuals with chronic conditions and that are added to standard primary care. The trial followed a parallel-group randomized controlled design with a delayed intervention for the control group and was conducted consecutively (ie, participants started sequentially rather than simultaneously). Participants were randomized to the intervention group and control group with an allocation ratio of 6:7. In the first 18 months, the intervention group received the intervention, whereas the control group received regular DMP care. After this interval, the control group was offered the intervention for 9 months. The primary outcome of the original trial was the number of hospital days during the year preceding the end of the intervention. Secondary outcomes covered three domains: (1) self-reported physical health status, (2) self-reported health-related behaviors, and (3) self-reported thoughts and feelings (ie, psychological variables).

### Patient and Public Involvement

No formal patient or public involvement took place in the design, conduct, or reporting of the trial.

### Changes to the Trial Protocol

In some aspects, the conduction of the trial deviated from the protocol. Not all peer support group meetings could be guided by members of the groups because not enough participants were willing to take on this role. Moreover, some peer support group meetings had to be held online because of the COVID-19 pandemic, and the feedback report only started with a delay due to organizational problems.

### Harms

In accordance with the trial protocol, harms and potential adverse events were monitored and documented during peer support group meetings and telephone coaching sessions. No adverse events or harms were reported.

### Sample Size

The sample size of the original trial was determined based on the primary outcome (ie, the number of hospital days in the last year of the intervention interval) and was powered to detect small effect sizes (Cohen *d*=0.2) with a significance level of .05 and a power of 0.80, resulting in a required sample size of 1001 participants, as detailed in the trial protocol [40]. No separate power calculation was conducted for this secondary analysis, which focused on a specific intervention component and on the intervention group alone.

### Investigated DHI

The browser-based web portal provided as part of the complete intervention (ie, the DHI investigated in this study) was developed based on the self-determination theory (SDT) [43]. Among other things, the SDT posits that individuals strive to fulfill the following 3 basic psychological needs: autonomy (eg, possession of choice and action scope), competence (eg, personal growth and skill development), and social relatedness (eg, relationship building and integration). The DHI was developed within this conceptual framework, with a particular focus on supporting autonomy and enhancing competence. The development was conducted in three stages: (1) a workshop, (2) content design by core teams, and (3) consolidation of content and posting on the web portal.

The aim of the workshop was to define the content, communication style, and visual design appropriate for the target group. This workshop was guided by an external digital agency based in Cologne. Participants of the workshop included members of the interdisciplinary project team (sports science, health science, psychology, and medicine), a practicing physician, and a potential study participant. Based on the workshop results, content and functions for the web portal were developed. Subsequently, the following four core teams were established (each for a different topic): (1) medicine, (2) physical activity, (3) nutrition, and (4) motivation. These core teams systematically developed the intervention content. In the final step, the developed content was consolidated and successively integrated into the DHI by a specific project team member.

**Figure 1.**
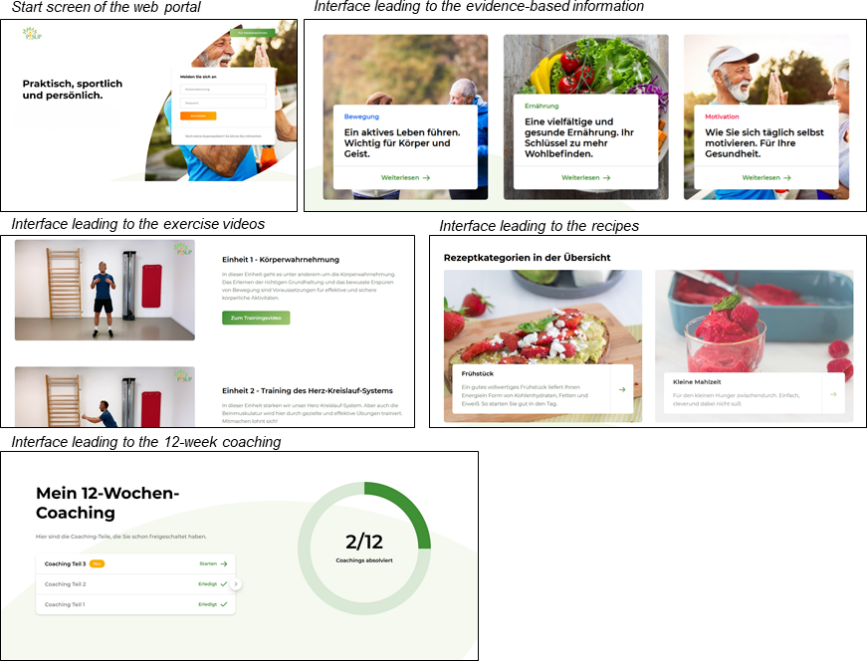
Screen capture images from the P-SUP (Personalized Self-management Support Program) web portal.

The web portal included the following: (1) evidence-based information regarding T2DM and CHD, physical activity, nutrition, and self-motivation; (2) exercise videos conducted by a sports therapist focusing on strength, endurance, coordination, and flexibility; (3) recipes for healthy food; (4) a motivational 12-week coaching program; and (5) a download area for nutritional help tools (Figure 1). Usability and visual design of the complete web portal were tested formatively during the development process with a cohort comparable to the final study sample. The 12-week coaching program was developed iteratively according to the Integrate, Design, Assess, and Share (IDEAS) framework [44-46]. For further details on the content and design of the web portal, see Multimedia Appendix 1.

The final web portal was made accessible to all participants of the intervention group by sending them a log-in identification number and a password by mail prior to the start of the study. In addition to the log-in credentials, they received a letter that included an explanation of the web portal contents, data protection information (eg, data storage, server location, and privacy), a step-by-step guide for the registration process, and contact details (email and telephone) for technical support. To maintain participant interest in the application, the web portal was updated with new content at irregular intervals. To accompany these updates and support user engagement, a total of 10 newsletters were distributed via email at regular intervals throughout the intervention period. These newsletters explained the different components of the P-SUP intervention and provided direct links to newly published content.

### Study Participants

The study participants of the complete RCT were recruited between February and October 2021 from 6 different regions in North Rhine-Westphalia, Germany, including Aachen, Bonn, Cologne, Düsseldorf, Essen, and Bünde, via general practitioners, newspapers, and online advertisements. General practitioners informed eligible patients about the intervention during routine DMP appointments and enrolled them directly. Individuals who responded to newspaper or online advertisements were enrolled in the study by the study team if they met the inclusion criteria. The inclusion criteria for patients were as follows: (1) being ≥18 years of age, (2) being enrolled in a DMP for T2DM and/or CHD [42], and (3) being a resident of North Rhine-Westphalia. The exclusion criteria were as follows: (1) insufficient German language skills, (2) severe cognitive or physical impairments, and (3) severe comorbidities. Those persons who were assigned to the intervention group and who were finally included in the analyses for examining the effectiveness of the complete intervention were also included in the secondary analysis presented in this article.

### Recruiting Sites and Personnel

Recruitment was conducted through general practitioners participating in the DMPs for T2DM and/or CHD. The eligibility for physician recruitment was limited to standard DMP accreditation. No additional eligibility criteria, training, or intervention delivery responsibilities were applicable, and no additional personnel involved in delivering or supporting the DHI were investigated in this secondary analysis.

### Data Collection and Measures

#### DHI Usage

DHI usage was determined based on server data from the web portal, covering the entire 18-month intervention period. Because of the consecutive nature of the study, these periods began in the period from April 2021 to February 2022. The collected data included information on log-ins and viewed content.

However, for some participants, the documentation of web portal usage did not start with an identified log-in. This indicated technical problems with the documentation. As a result, the log-ins, which were meant to be the basic units of DHI usage, could not be determined via the log-in data. Instead, log-ins were partly reconstructed based on timestamps of viewed content. A new log-in was assumed whenever there was a gap of at least 2 hours between 2 consecutive content views. This threshold was deliberately set longer than the 30 or 60 minutes recommended in previous literature [47,48], because certain types of content on the web portal, such as exercise videos, lasted around 30 minutes. Thus, if a participant logged into the web portal and subsequently watched 1-2 videos, the activity following this should still be attributed to the same session.

Uptake was operationalized as the incidence of at least one log-in, whereas frequency was operationalized as the total number of log-ins minus the first one. The first log-in was subtracted because the frequencies were analyzed to obtain information about processes that occur after the first log-in. As a sensitivity analysis, all analyses referring to frequencies were also performed with log-ins defined using 30- and 60-minute intervals.

All variables determined for operationalizing usage only reflected direct use of the DHI. Indirect use was possible by downloading exercise videos or healthy food recipes and using them offline. This shifted some web portal activities from tracked online sessions to unobserved offline use and thus likely led to an underestimation of usage frequency.

#### Possible Determinants of DHI Usage

Possible determinants of DHI usage were selected from two sources: (1) register data from the organization unit coordinating the whole project, and (2) data from the baseline survey conducted prior to the start of the intervention. The register data were collected and updated from January 2021 to April 2024. The baseline survey was conducted from February to December 2021. The criterion for selecting variables from these 2 sources was that these variables had already been considered as possible determinants in previous research regarding the determinants of DHI usage.

#### Sociodemographic Variables

Sociodemographic characteristics included age, gender, and educational attainment. Age and gender were assessed via both the register data and the baseline survey. Educational attainment was only assessed via the baseline survey. In this survey, participants were asked whether they had the qualification required to attend a higher education institution, such as a university of applied sciences or an ordinary university. Those who answered “yes” were classified as having a higher level of educational attainment, while those who answered “no” were classified as having a lower level. Data for age and gender were available for all participants. Missing data for education were multiply imputed by producing 50 different datasets using the multiple imputation procedure provided as standard by SPSS (IBM Corp).

#### Psychological Variables

Psychological variables were solely assessed via the baseline survey. These variables can be assigned to two different categories: (1) general psychological variables, and (2) health-related psychological variables.

Five general psychological variables included the “big five” (ie, openness, conscientiousness, extraversion, agreeableness, and neuroticism). Three further general psychological variables included loneliness, depression, and anxiety. The “big five” variables were assessed using the Big Five Inventory-10 items (BFI-10) [49], which consists of 10 items, with each dimension addressed by 2 items. Loneliness was assessed using the 6-item short version of the revised University of California Los Angeles (UCLA) Loneliness Scale [50,51]. Depression and anxiety were assessed using the Patient Health Questionnaire-4 items (PHQ-4). The PHQ-4 is a 4-item self-report questionnaire that measures depression using the first 2 items and anxiety using the last 2 items [52].

The health-related psychological variables were health literacy, patient activation, and intentions regarding physical activity or healthy nutrition. Health literacy was assessed using the 5-item Brief Health Literacy Screen (BHLS) [53], and patient activation was evaluated using the Patient Activation Measure-13 (PAM13-D) [54,55]. Each of the 2 intention variables was assessed using 1 item with a 7-category scale. Physical activity was operationalized as engaging in at least 150 minutes of moderate or vigorous physical activity per week (“Do you intend to engage in at least two-and-a-half hours of moderate or vigorous physical activity per week in the coming months?”), and healthy nutrition was operationalized as performing at least five of nine items of healthy nutrition behavior (“Do you intend to follow at least five of the dietary behaviors described above in the coming months?”) listed in a self-constructed questionnaire (Multimedia Appendix 2).

Missing data for psychological variables were multiply imputed by producing 50 different datasets using the multiple imputation procedure provided as standard by SPSS. Except for the PAM13-D, all variables based on more than one item were constructed by summing up the scale values assigned to the different answer categories, with all answer categories coded with equal distances between all adjacent categories. For the PAM13-D, the sums determined in this way were additionally transformed into scale values obtained by Rasch scaling [56] (Multimedia Appendix 3).

All psychological variables were standardized via a linear transformation, with 0 being the lowest and 1 the highest value that could be obtained with the respective measurement instrument. This standardization was performed because all these variables possess at best an interval scale level, and thus, the corresponding measurement values have no absolute meaning but only a meaning relative to 2 reference points. For all variables, the boundaries of the corresponding measurement instrument constitute the best available reference points, and coding these reference points with 0 and 1 makes it possible to compare the magnitudes of the effects of these variables on DHI usage. All variables coded this way were pooled according to the denomination applied for the respective variable (ie, 1 is the highest level of extraversion, agreeableness, openness, conscientiousness, neuroticism, loneliness, depression, anxiety, patient activation, health literacy, intention regarding physical activity, or intention regarding healthy nutrition).

### Statistical Analysis

Patient characteristics were documented based on complete data. This was put into practice separately for nonusers and users, as well as for all investigated persons together. Gender and education were described using absolute and relative frequencies; all other variables were described using means and SDs. To provide a clear overview of overall usage of the web portal, the 18-month intervention period (78 weeks) was divided into 13 intervals of 6 weeks each. This segmentation was chosen because 78 is divisible by 13, allowing for consistent time intervals across the entire period. For each 6-week interval, user activity was described using relative and absolute frequencies. Absolute frequencies included (1) the absolute frequency of log-ins per interval; (2) the absolute frequency of users per interval; and (3) the absolute frequency of first log-ins per interval. Relative frequencies included (1) the relative frequency of log-ins per interval, expressed as a percentage of all log-ins across all intervals; (2) the relative frequency of users per interval, expressed as a percentage of all user occurrences across all intervals; and (3) the relative frequency of first log-ins per interval, expressed as a percentage of all first log-ins across all intervals.

All further analyses were conducted with missing values being multiply imputed. In response to comments raised during the review process, a test for data missing completely at random (MCAR) was performed [57]. However, the results of this test have no implications for the interpretation of the results of the further analyses, because statistical analyses based on multiply imputed data are unbiased as long as the much less restrictive condition of MAR (ie, data missing at random) is fulfilled [58]. However, for this condition, no feasible statistical test presently exists.

The analyses regarding uptake and frequency were conducted with different samples. For uptake, these were all members of the intervention group, while for frequency, these were only those who had used the web portal at least once (ie, users). In both cases, three different analyses were performed: (1) an analysis of the correlations between the variables investigated as possible determinants; (2) simple regression analyses for all possible determinants, with either uptake or frequency as a criterion; and (3) a multiple regression analysis with all possible determinants entered as predictors in a common model, with either uptake or frequency as a criterion. If the usage variable was uptake, logistic regression was applied, while if the usage variable was frequency, negative binomial regression with data-based (robust) SEs was applied. The latter procedure was chosen because negative binomial regression is specifically designed for count variables and because data-based SEs are not based on a specific model, so that inference statistical tests based on these errors cannot be compromised by invalidity of the presupposed model [59].

Several different statistics were computed in connection with the regression analyses. These were regression coefficients, corresponding 95% CIs, and corresponding *P* values for all regression analyses. In logistic regression, Nagelkerke pseudo *R*^2^ was used to assess how well the determinants explain the actual values. In negative binomial regression, the proportion of explained variance was used for this purpose. For negative binomial regression, the dispersion parameter was also computed. This parameter refers to the variance of the actual values that are predicted by the same predicted value. If this variance is equal to or lower than the predicted value, the dispersion parameter is 0. Otherwise, this parameter is larger than 0, with larger dispersion parameters indicating larger variances. To investigate how the different predictor variables interfere with each other when regression coefficients in multiple regression are estimated, variance inflation factors were computed for both multiple regressions. These factors are equal to 1 if there is no interference at all, and they increase with increasing interference. With a variance inflation factor larger than 5, the estimates of the regression coefficients are not very trustworthy.

Inflation of α errors due to multiple testing was controlled differently for simple and multiple regression. For simple regression, the approach of Benjamini and Hochberg was applied [60]. In this approach, a specific comparator is computed for each *P* value. For this purpose, the *P* values for the different tests are arranged in ascending order. The comparator for each *P* value is as follows:


(1) $${comparator}_{i}=\frac{i}{m}\alpha$$

where *i* is the test number in ascending order, *m* is the number of tests, and α is the significance level applied for an individual test. The largest *i* for which the *P* value is smaller than the comparator is identified, and all tests with an index smaller than or equal to this *i* are considered to be significant. In multiple regression, the final decision regarding the significance was based on the result of the significance test of the complete regression model. If this test yielded a significant result, all significant results of tests regarding the individual regression coefficients were considered as actually significant; otherwise, none of the individual test results were considered as significant.

To compare the regression models for uptake and frequency, the rank correlation between the *P* values was computed.

All data were analyzed using IBM SPSS Statistics version 29.

### Ethical Considerations

Ethical approval for the complete RCT was obtained from the Ethics Committee of the Medical Faculty of the University Hospital Cologne in July 2020 (20-1155). All patients provided written informed consent prior to participation in the RCT. This consent also covered the use of pseudonymized data for secondary analyses without requiring additional consent. Patient data were stored within three separate organizational units at the University Hospital Cologne: (1) Trust Center, which held address data linked to study identification numbers; (2) Data Warehouse, which held evaluation data linked to study identification numbers; and (3) Multiple Pseudonym Assignment Unit, which maintained the assignment list linking personal and study identification numbers. These units were strictly separated both digitally and in terms of personnel, ensuring confidentiality and preventing reidentification. Participants received no financial incentives.

No images in the article or supplementary materials allow for the identification of individual participants. Any visible human faces have been deliberately blurred to ensure anonymity, or the faces originate from commercially available stock images and do not depict study participants. No consent from identifiable individuals was therefore required.

## Results

### General Information

**Figure 2.**
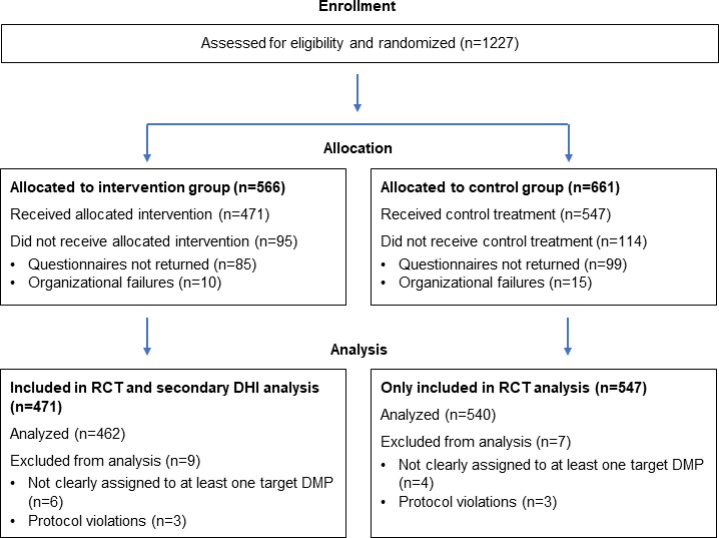
CONSORT (Consolidated Standards of Reporting Trials) flow diagram. DHI: digital health intervention; DMP: disease management program; RCT: randomized controlled trial.

A total of 1227 patients were enrolled in the RCT, with 566 patients allocated to the intervention group and 661 patients allocated to the control group. Ultimately, 1002 patients were included in the RCT analyses (intervention group: n=462; control group: n=540; Figure 2). The analyses presented here are restricted to the 462 participants allocated to the intervention group with available baseline questionnaire data and register data at the start of the intervention. The flow diagram in Figure 2 follows the structure recommended by CONSORT (Consolidated Standards of Reporting Trials) 2025 [61]; however, only the elements relevant to this secondary analysis are addressed in the flow diagram. The CONSORT checklist is presented in Checklist 1. Follow-up assessments and analyses of primary and secondary outcomes were conducted as part of the trial but are not relevant to the analyses reported here and will be presented in the effectiveness evaluation in a separate publication.

Among the data included in the secondary analysis, an average of 2.2% (range 0.7%-4.8%) of data were missing. MCAR was not fulfilled (*P*=.008). The mean participant age at the time of the survey was 64.8 (SD 10.2) years. Moreover, 49.8% (230/462) of participants were female, 38.6% (170/462) had a university entrance qualification, and 43.1% (199/462) had at least one log-in to the web portal (Table 1). Participants in the last group were classified as users.

**Table 1.** Patient characteristics^a^.

Variable	Nonusers		Users		Total	
	n^b^	Statistics	n^b^	Statistics	n^b^	Statistics
Sociodemographic variable						
Age (years), mean (SD)	263	65.4 (11.0)	199	64.0 (9.2)	462	64.8 (10.2)
Female gender^c^, n (%)	263	125 (47.5)	199	92 (46.2)	462	230 (49.8)
Higher education, n (%)	248	81 (32.7)	192	89 (46.4)	440	170 (38.6)
Psychological variables^d^						
General psychological variables, mean (SD)						
Extraversion	254	0.60 (0.24)	195	0.60 (0.26)	449	0.60 (0.25)
Agreeableness	258	0.56 (0.21)	195	0.56 (0.19)	453	0.56 (0.20)
Openness	259	0.55 (0.26)	195	0.62 (0.24)	454	0.58 (0.25)
Conscientiousness	254	0.70 (0.21)	195	0.69 (0.20)	449	0.70 (0.21)
Neuroticism	261	0.49 (0.23)	195	0.48 (0.23)	456	0.49 (0.23)
Loneliness	254	0.32 (0.28)	194	0.35 (0.27)	448	0.33 (0.28)
Depression	255	0.24 (0.23)	198	0.24 (0.24)	453	0.24 (0.24)
Anxiety	255	0.23 (0.25)	198	0.22 (0.23)	453	0.23 (0.24)
Health-related psychological variables, mean (SD)						
Patient activation	254	0.67 (0.16)	197	0.65 (0.13)	451	0.66 (0.15)
Health literacy	250	0.70 (0.21)	196	0.73 (0.18)	446	0.71 (0.20)
Intention–physical activity	257	0.79 (0.27)	198	0.89 (0.16)	455	0.84 (0.23)
Intention–healthy nutrition	261	0.78 (0.25)	198	0.88 (0.15)	459	0.82 (0.22)

a Patients are members of the intervention group of the randomized controlled trial conducted within the P-SUP (Personalized Self-Management Support Program) project. They were enrolled in disease management programs for type 2 diabetes and/or coronary heart disease and were residing in North Rhine-Westphalia, Germany. Data were collected during the baseline assessment of the P-SUP project.

b Number of persons with complete data for the respective variable.

c None of the participants reported their gender as diverse.

d For better comparability, all psychological variables were scaled to a range from 0 to 1. Higher values indicate a higher expression of the construct.

**Figure 3.**
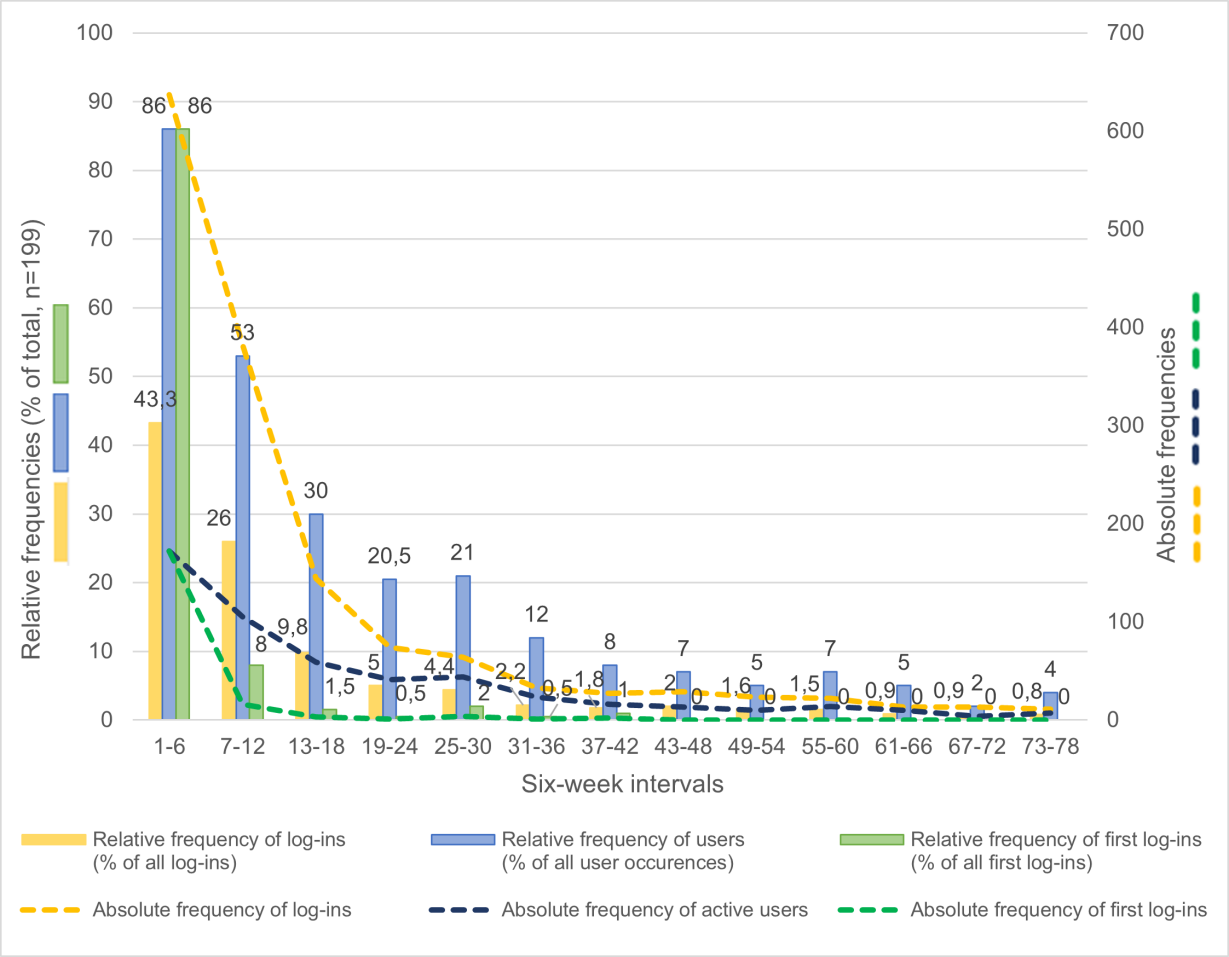
User activity.

Among users, 86.4% (172/199) had their first log-in within the first 6 weeks of the complete intervention period. The final first log-in was recorded in week 39. The total number of log-ins was 637 within these first 6 weeks. In the following 6-week intervals, both the number of persons using the web portal and the total number of log-ins decreased almost continuously (Figure 3). With the first log-in included, participants classified as users visited the web portal on average 7.4 (SD 8.5; range 1‐50) times, if log-ins were determined using an interval of 120 minutes between 2 subsequent activities on the web portal. For intervals of 60 and 30 minutes, the corresponding values were 7.7 (SD 8.8; range 1‐52) times and 8.2 (SD 9.5; range 1‐59) times, respectively.

### Uptake

Many of the variables taken as possible determinants for uptake correlated moderately with each other. This applied especially to loneliness, depression, and anxiety, as well as to intentions regarding physical activity and healthy nutrition (Multimedia Appendix 4). Nevertheless, the largest inflation factor was smaller than 3 (Table 2). Accordingly, all possible determinants could be included in the multiple regression.

**Table 2.** Simple and multiple regression analyses for predictors of uptake^a^.

Variable	Simple regression analysis^b^			Multiple regression analysis^b^		
	B^c^ (95% CI)	*P* value	NK^d^	B^c^ (95% CI)	*P* value	VIF^e^
Sociodemographic variables						
Age in years	−0.01 (−0.03 to 0.00)	.12	0.006	−0.00 (−0.02 to 0.02)	.90	1.14
Female gender	−0.25 (−0.62 to 0.12)	.18	0.005	−0.30 (−0.73 to 0.12)	.16	1.15
Higher education	0.56 (0.18 to 0.95)	.004^f^	0.024	0.29 (−0.15 to 0.73)	.20	1.14
Psychological variables^g^						
General psychological variables						
Extraversion	−0.05 (−0.81 to 0.70)	.89	0.000	−0.08 (−1.00 to 0.84)	.87	1.27
Agreeableness	−0.18 (−1.09 to 0.73)	.70	0.000	0.02 (−1.01 to 1.06)	.97	1.09
Openness	1.08 (0.33 to 1.83)	.005^f^	0.024	0.81 (−0.06 to 1.67)	.07	1.18
Conscientiousness	−0.31 (−1.21 to 0.58)	.49	0.001	−0.03 (−1.11 to 1.04)	.95	1.23
Neuroticism	−0.14 (−0.94 to 0.67)	.74	0.000	−0.05 (−1.06 to 0.96)	.92	1.35
Loneliness	0.34 (−0.33 to 1.01)	.32	0.003	0.54 (−0.43 to 1.51)	.27	1.76
Depression	0.07 (−0.72 to 0.85)	.87	0.000	0.08 (−1.33 to 1.48)	.92	2.64
Anxiety	−0.08 (−0.85 to 0.68)	.83	0.000	−0.38 (−1.83 to 1.07)	.61	2.96
Health-related psychological variables						
Patient activation	−1.17 (−2.48 to 0.14)	.08	0.010	−3.20 (−4.95 to −1.46)	<.001^f^	1.36
Health literacy	1.05 (0.08 to 2.03)	.03^f^	0.014	1.01 (−0.26 to 2.29)	.12	1.43
Intention–physical activity	2.28 (1.30 to 3.26)	<.001^f^	0.071	1.86 (0.74 to 2.97)	.001^f^	1.23
Intention–healthy nutrition	2.30 (1.30 to 3.31)	<.001^f^	0.067	2.22 (1.00 to 3.44)	<.001^f^	1.34

a Patients are members of the intervention group of the randomized controlled trial conducted within the P-SUP (Personalized Self-Management Support Program) project. They were enrolled in disease management programs for type 2 diabetes and/or coronary heart disease and were residing in North Rhine-Westphalia, Germany. Data were collected during the baseline assessment of the P-SUP project.

b Simple and multiple regression analyses performed on data after multiple imputation (n=462).

c B: regression coefficient B.

d NK: Nagelkerke pseudo *R*2.

e VIF: variance inflation factor.

f Significant value.

g For better comparability, all psychological variables were scaled to a range from 0 to 1. Higher values indicate a higher expression of the construct.

In the simple regression analyses, education, openness, health literacy, intention regarding physical activity, and intention regarding healthy nutrition had statistically significant effects on uptake (education: *P*=.004; openness: *P*=.005; health literacy: *P*=.03; intention-physical activity: *P*<.001; intention-healthy nutrition: *P*<.001; Table 2). In all 5 cases, these effects were positive, with the effects for the 2 intention variables being the largest. After controlling for multiple testing according to Benjamini and Hochberg [60], the effect for health literacy disappeared, but the effects for the other 4 variables with significant effects in the individual tests remained. In the multiple regression analysis, the pattern of statistically significant effects changed. Education (*P*=.20), openness (*P*=.07), and health literacy (*P*=.12) no longer had statistically significant effects. Instead, patient activation had a strong statistically significant negative effect on uptake (*P*<.001), that is, persons with high patient activation were less likely to use the web portal. The intentions regarding physical activity and healthy nutrition remained statistically significant positive predictors (intention-physical activity: *P*=.001; intention-healthy nutrition: *P*<.001). However, the positive effects of these 2 variables were smaller than the negative effect of patient activation. The complete model used in the multiple regression analysis had high statistical significance (*P*<.001; Table 2). This indicates that the statistically significant effects found for 3 of the 15 variables cannot simply be explained by a higher chance of α errors due to multiple testing. Nagelkerke pseudo *R*^2^ for the complete model was 0.18.

### Frequency

If the correlations between the possible determinants are only computed for participants who used the web portal at least once, the pattern of correlations would be very similar to the pattern of correlations for all investigated persons. Many of the variables analyzed were moderately correlated with each other. Again, the highest correlations were among loneliness, depression, and anxiety. However, the correlation between the intentions regarding physical activity and healthy nutrition decreased (Multimedia Appendix 5). Notwithstanding the moderate correlations, the largest inflation factor was smaller than 3, similar to the finding in the analyses for uptake (Table 3). Accordingly, all possible determinants could be included in the multiple regression.

**Table 3.** Simple and multiple regression analyses for predictors of frequency^a^.

Variable	Simple regression analysis^b^					Multiple regression analysis^b^			
	B^c^ (95% CI)		*P* value	DP^d^	PEV^e^	B^c^ (95% CI)		*P* value	VIF^f^
Sociodemographic variables									
Age in years	0.03 (0.01 to 0.05)		.005^g^	1.66	0.024	0.02 (0.00 to 0.04)		.03^g^	1.19
Female gender	0.12 (−0.25 to 0.49)		.53	1.72	0.002	0.25 (−0.09 to 0.59)		.15	1.25
Higher education	0.01 (−0.36 to 0.39)		.94	1.73	0.000	−0.02 (−0.36 to 0.33)		.93	1.15
Psychological variables^h^									
General psychological variables									
Extraversion	−0.41 (−1.16 to 0.33)		.28	1.71	0.004	−0.63 (−1.37 to 0.12)		.10	1.43
Agreeableness	−0.42 (−1.35 to 0.51)		.37	1.72	0.004	−0.65 (−1.43 to 0.14)		.11	1.21
Openness	−0.14 (−0.88 to 0.60)		.72	1.72	0.000	0.04 (−0.64 to 0.72)		.91	1.15
Conscientiousness	1.09 (0.25 to 1.93)		.01^g^	1.66	0.039	1.00 (0.16 to 1.84)		.02^g^	1.34
Neuroticism	0.37 (−0.40 to 1.14)		.35	1.72	0.003	0.50 (−0.32 to 1.33)		.23	1.53
Loneliness	−0.62 (−1.26 to 0.01)		.06	1.69	0.020	−0.64 (−1.42 to 0.15)		.11	1.94
Depression	−0.89 (−1.58 to −0.19)		.01^g^	1.68	0.015	−0.74 (−1.64 to 0.17)		.11	2.58
Anxiety	−0.24 (−1.05 to 0.58)		.57	1.72	0.001	0.60 (−0.52 to 1.72)		.29	2.88
Health-related psychological variables									
Patient activation	1.25 (−0.12 to 2.62)		.07	1.69	0.018	0.52 (−0.95 to 1.98)		.49	1.57
Health literacy	0.35 (−0.78 to 1.48)		.55	1.72	0.002	0.09 (−1.08 to 1.25)		.89	1.56
Intention–physical activity	−0.19 (−1.12 to 0.75)		.69	1.72	0.001	−0.63 (−1.49 to 0.22)		.15	1.17
Intention–healthy nutrition	0.49 (−0.82 to 1.79)		.47	1.72	0.002	0.48 (−0.61 to 1.57)		.39	1.19

a Patients are members of the intervention group of the randomized controlled trial conducted within the P-SUP (Personalized Self-Management Support Program) project. They were enrolled in disease management programs for type 2 diabetes and/or coronary heart disease and were residing in North Rhine-Westphalia, Germany. Data were collected during the baseline assessment of the P-SUP project.

b Simple and multiple regression analyses performed on data after multiple imputation (n=199).

c B: regression coefficient B.

d DP: dispersion parameter.

e PEV: proportion of explained variance.

f VIF: variance inflation factor.

g Significant value.

h For better comparability, all psychological variables were scaled to a range from 0 to 1. Higher values indicate a higher expression of the construct.

The results of the regression analyses were virtually the same for all 3 intervals applied for identifying new log-ins. Therefore, only the results for the 120-minute interval have been reported. These results differed strongly from those for uptake (Table 3). In the simple regression analyses, only 3 of the 15 variables had statistically significant effects (ie, age: *P*=.005; conscientiousness: *P*=.01; and depression: *P*=.01). The first 2 variables related positively to frequency, whereas there was a negative effect for depression. However, after controlling for multiple testing according to Benjamini and Hochberg [60], all 3 effects disappeared. In the multiple regression analysis, there were effects for age (*P*=.03) and conscientiousness (*P*=.02), whereas the effect for depression was no longer observed (*P*=.11; Table 3). However, in contrast to the complete multiple regression model for predicting uptake, the complete multiple regression model for predicting frequency was not statistically significant (*P*=.07). Accordingly, whether age and conscientiousness actually affect frequency is questionable. The dispersion parameter for the complete model was 1.48, and the proportion of explained variance was 0.16.

### Comparison Between Uptake and Frequency

The comparison of the multiple regression models for uptake and frequency showed that the rank order of the *P* values differed markedly between uptake and frequency. The correlation between the rank orders was −0.50, with a statistically significant deviation from 0 (*P*=.009).

## Discussion

### Principal Findings

The main finding of this study is that uptake and frequency of use are influenced in different ways by the variables considered as potential determinants. In simple regression analyses, uptake of the web portal was positively associated with higher education, openness, health literacy, and intentions regarding physical activity and healthy nutrition; however, the effect for health literacy did not remain significant after controlling for multiple testing. In the multiple regression model, uptake was primarily explained by strong intentions regarding physical activity and healthy nutrition, alongside a significant negative association with patient activation. In contrast, frequency of use showed a different pattern. In simple regression analyses, frequency was positively associated with age and conscientiousness and negatively associated with depression; however, in the multiple regression model, only age and conscientiousness remained associated. After controlling for inflation of the α error, none of the associations for frequency of use remained statistically significant, and the multiple regression model for frequency was not significant.

The observation that the multivariate regression model has a strong effect for uptake but no statistically significant effect for frequency may be partly attributable to the smaller sample size available for the frequency analyses. As higher values of Nagelkerke pseudo *R*^2^ are more difficult to obtain than higher values of proportions of explained variance for continuous variables [62], the comparison of these 2 statistics suggests that the set of variables investigated in this study has a stronger impact on uptake than on frequency of use. A further difference between the 2 analyses concerns the rank order of the *P* values for the 2 multiple regressions, which deviated markedly between uptake and frequency. This strongly indicates that the considered variables affect uptake and frequency in very different ways. This demonstrates that both components of usage are distinct and should be investigated and interpreted separately from each other.

### Uptake

In this study, intentions had a very strong positive association with uptake. This finding is supported by research on behavior change, which emphasizes the central role of intention in bridging the gap between attitudes and actual behavior [63]. According to the Theory of Planned Behavior [64], intention is viewed as the proximal determinant of behavior. According to this, people are more likely to perform a behavior if they strongly intend to do so. In a previous study that most closely resembles this study, Voncken-Brewster et al [26] examined a web-based self-management intervention for individuals with or at high risk for chronic obstructive pulmonary disease (COPD). In a common multiple regression model, they considered age, gender, education, and intention to change behavior alongside clinical factors such as COPD status, smoking status, physical activity level, and dyspnea status and found no statistically significant effects. In contrast, this study identified a strong positive association between intention and uptake. The difference between the 2 results might be explained by the fact that both studies refer to different kinds of intentions. In the study by Voncken-Brewster et al [26], intention referred to a change in a specific behavior, while in this study, intention referred to the performance of a specific behavior. The latter seems to be a strong motivating factor for uptake, whereas the former does not show the same effect.

In this study, patient activation showed a strong negative association with uptake. This contrasts with previous findings, where either a positive association [37] or no effect was reported [36]. A central assumption is that higher patient activation is associated with greater engagement in health-promoting behaviors [65]. However, DHIs are not necessarily health-promoting, and the DHI investigated in this study may provide little additional value for individuals who already engage in the targeted behaviors. Highly activated individuals may therefore be less inclined to use such interventions. In contrast, less activated individuals in this study may have been more likely to perceive the DHI as beneficial, given that individuals with lower activation levels are more likely to experience poorer clinical outcomes and higher rates of hospitalization [65], and thus have greater potential to benefit from additional self-management support. When initial use does not require immediate behavioral change, such tools may represent an accessible entry point into health-related activation. This suggests that the relationship between patient activation and DHI uptake may depend on perceived relevance and expected benefit. Differences in the added value of the DHIs investigated across studies may therefore explain the divergent findings. Whether this mechanism accounts for the negative association observed in this study remains a question for future research.

In this study, the effect of openness was only seen in the simple analysis and not in the multiple regression analysis. This finding might be unexpected, as individuals with high openness are generally more willing to explore unfamiliar tools and information sources [66]. Moreover, in a previous study by Su et al [29], where a smartphone-based app for improving self-management in people with T2DM was examined, openness had a statistically significant positive effect on uptake. One possible explanation for this discrepancy is that Su et al [29] did not include an intention variable in their multiple regression analysis. Given the strong and proximal role of intention in predicting behavior, intention may represent a more proximal and stronger determinant of uptake, thereby attenuating the independent effect of more distal personality traits, such as openness, when both variables are considered simultaneously. This interpretation is further supported by evidence suggesting that openness primarily influences behavior by moderating the intention-behavior relationship rather than exerting a strong independent effect. Openness has been shown to enhance the predictive strength of intention for behavior, while its direct association diminishes once intention is accounted for [67].

Taken together, the findings for uptake suggest that individuals who either have low intention to engage in health-promoting behaviors or consider their current levels of physical activity and healthy nutrition to be sufficient may not be reached by such interventions. To increase uptake among individuals with low intention with regard to the targeted behaviors, this intention may first need to be developed. This could be achieved through targeted outreach strategies, motivational inputs that emphasize the relevance and potential benefits of the intervention, or integration into routine care settings such as general practice consultations [68]. In particular, endorsement by trusted health care professionals may help legitimize the intervention and lower initial barriers to engagement [69]. Highly activated individuals might benefit from DHIs that go beyond structured guidance and motivational support by providing tools that support the optimization of routines, monitoring of progress, and maintenance of long-term health-promoting behaviors. In contrast, less activated patients may benefit from low-threshold DHIs that provide consolidated, easily accessible information and basic guidance. Such interventions may reduce initial barriers to engagement by requiring little effort or commitment at the outset and may serve as an accessible starting point for health-related activation.

### Frequency

Without controlling for multiple testing, both simple and multiple regression analyses showed statistically significant positive effects of age and conscientiousness on frequency of use. After controlling for multiple testing, none of these effects remained statistically significant in any of the regression analyses. The following considerations regarding age and conscientiousness are therefore exploratory and are intended to contextualize observed tendencies only.

The tendency regarding age found in this study is consistent with findings reported by Goyal et al [24], who examined a smartphone-based app for the assessment and prevention of heart disease. In that study, a core feature of the app was the proposal of health-related activities, referred to as challenges, and the frequency of use was operationalized as the number of challenges completed within the first 30 days of app use. An explanation of this effect or tendency might be that health-related goals tend to increase over a person’s life course [70,71] because health becomes a more central concern due to increasing functional limitations and health issues [72]. Furthermore, older individuals may particularly appreciate DHIs that bundle relevant information in an easily accessible and user-friendly platform and allow self-paced engagement [73]. The lower uptake tendency among younger individuals may reflect different usage preferences rather than a lack of interest in health topics. Younger individuals may be more likely to access health-related information through multiple digital channels and may prefer more flexible or interactive formats due to their higher digital health literacy [74]. Further studies should investigate whether different types of DHIs are preferentially used by different age groups, which could inform more age-sensitive design and implementation strategies.

There was no gender effect in this study. This contrasts with findings from previous studies showing that women are generally more likely than men to engage in health-promoting behavior [75-77] and that gender influences DHI use [24,78]. However, the direction of this gender effect was not specified in the study by Goyal et al [24], whereas Rising et al [78] reported higher DHI use among women. One explanation for the absence of a gender effect in this study is that prior studies used substantially larger sample sizes (n=69,952 and n=6789), which increased the likelihood of detecting gender differences. A further reason might be the measurement of usage. Goyal et al [24] assessed usage based on the completion of real-life health challenges, such as engaging in physical activity or preparing healthy meals, while this study focused on direct interaction with the DHI. Rising et al [78] relied on self-reported patterns of digital health use derived from questionnaire data, distinguishing users based on ownership and subjective use of smartphones, apps, and tracking devices rather than on actual interaction with a specific DHI. These differences in how usage is operationalized may lead to divergent findings regarding gender effects.

As no statistically significant effects on frequency of use were observed after controlling for multiple testing, no practical implications were derived from these findings.

### Limitations

The study has 5 main limitations regarding the interpretability of the results. First, the selection of potential determinants was limited to variables available in the register and survey data. As the survey did not assess participants’ access to digital devices or their level of digital health literacy [79], the results will presumably not cover all possible determinants of usage. Second, the operationalization of the frequency of use was a limitation. The participants had the option to download the exercise videos provided within the DHI. Since neither the number of downloads nor the frequency of offline usage could be tracked, actual usage may have been underestimated, potentially affecting the results. Third, the study was embedded in a complex, multicomponent intervention. The other components may have interacted with the effect of the web portal on DHI usage. Fourth, just as in all other studies addressing the relationship between person-related variables and DHI use, the variables examined might correlate with other variables that are not examined, and these variables, in turn, might be the actual determinants of DHI usage. Fifth, the investigations presented here refer to a very specific DHI and target group. All participants were enrolled in a German DMP and recruited in a specific region in Germany, where digital infrastructure, care pathways, and culture may differ from those in other regions or countries. Thus, usage patterns and determinants of usage may not be generalizable to other DHIs, other persons, or other settings.

### Conclusion

This study differs from previous studies in jointly examining the determinants of uptake and frequency of use for the same DHI within the same context and sample. It contributes to a more differentiated understanding of DHI use by demonstrating that uptake and frequency of use are influenced by distinct patterns of determinants. Uptake was primarily driven by strong behavioral intentions, whereas associations with frequency of use were less robust after adjustment for multiple testing. These findings suggest that initial adoption and sustained engagement represent related but analytically and practically distinct processes. Consequently, strategies aimed at promoting uptake may need to focus on strengthening health-related intentions, while efforts to foster continued use may require different, potentially more context-sensitive approaches. Applying the approach of this study to other DHIs and settings may support more targeted, effective, and equitable digital health development and implementation.

## Supplementary material

10.2196/80895 Multimedia Appendix 1 Web portal design and content.

10.2196/80895 Multimedia Appendix 2 Nutritional behavior questionnaire

10.2196/80895 Multimedia Appendix 3 Rasch scaling.

10.2196/80895 Multimedia Appendix 4 Correlation analysis for uptake.

10.2196/80895 Multimedia Appendix 5 Correlation analysis for frequency.

10.2196/80895 Checklist 1 CONSORT checklist

## Abbreviations

BFI-10: Big Five Inventory-10 items
BHLS: Brief Health Literacy Screen
CHD: coronary heart disease
CONSORT: Consolidated Standards of Reporting Trials
COPD: chronic obstructive pulmonary disease
DHI: digital health intervention
DMP: disease management program
IDEAS: Integrate, Design, Assess, and Share
MCAR: missing completely at random
P-SUP: Personalized Self-management Support Program
PAM13-D: Patient Activation Measure-13
PHQ-4: Patient Health Questionnaire-4 items
RCT: randomized controlled trial
SDT: self-determination theory
T2DM: type 2 diabetes mellitus
UCLA: University of California Los Angeles
