# Supplementary material for: Determinants of the Uptake and Frequency of Use of a Web Portal Digital Health Intervention in Patients With Type 2 Diabetes and/or Coronary Heart Disease: Secondary Analysis of a Randomized Controlled Trial
Source: J Med Internet Res. 2026 Mar 25;28:e80895. doi: 10.2196/80895 (PMC13016439; doi:10.2196/80895)
Supplement: Multimedia Appendix 1 [file jmir-v28-e80895-s001.doc]

**Web portal content**

**Start screen**


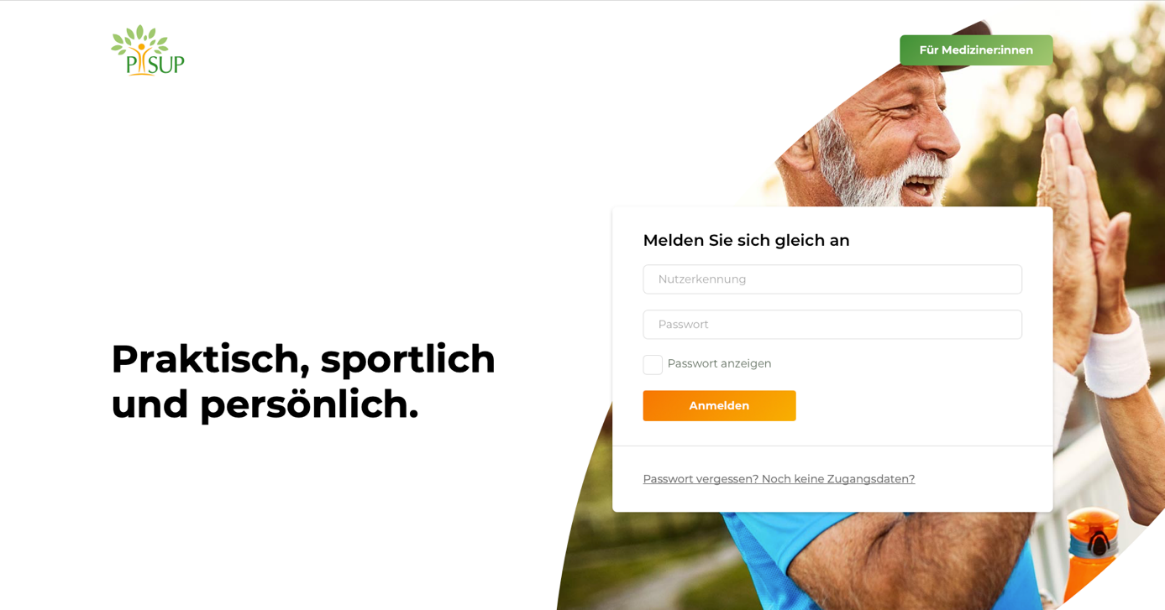
Patients could access the web portal using a user ID and password provided to them after enrolment in the programme (see Figure S1).

Figure S1: Start screen of the web portal

**Evidence-based information**

*Interactive eLearning Modules*

Knowledge about type 2 diabetes mellitus (T2DM) and coronary heart disease (CHD) was delivered through interactive eLearning courses. The content primarily covered disease definitions, along with therapy and treatment options. Each eLearning unit concluded with a quiz and a summary of key points.

*Blog Articles*


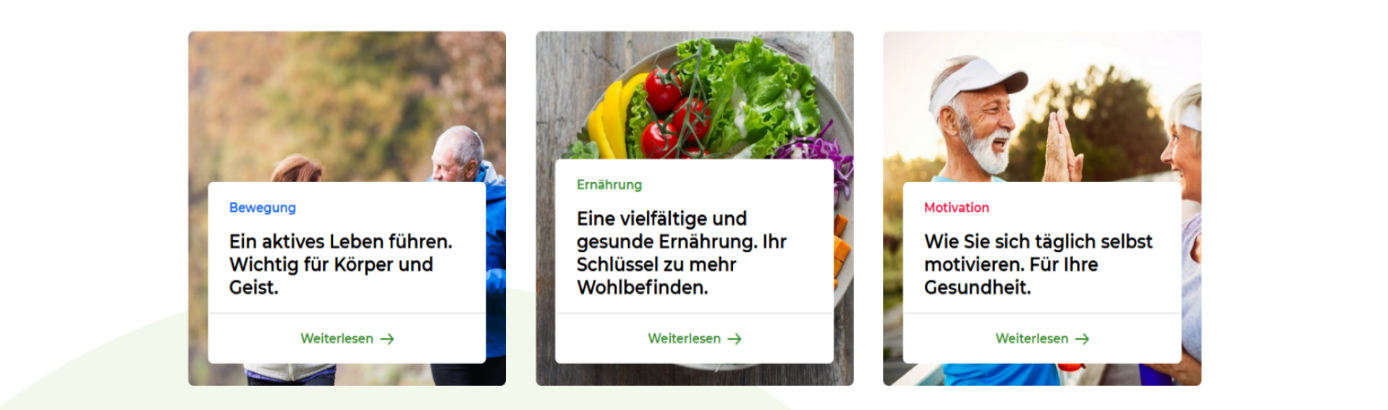
Participants had access to blog articles on physical activity, nutrition, motivation and medical information related to T2DM and CHD (see Figure S2). The articles were published sequentially over the course of the programme.

Figure S2: Interface leading to the blog articles

The following articles on physical activity, nutrition, motivation and medical information were accessible:

Table S1: Topics of the blog articles

| **Physical Activity** | **Nutrition** | **Motivation** | **Medical Information** |
| --- | --- | --- | --- |
| The Link Between Physical Activity and Health | Fat Is Not Just Fat | Health Changes – How Does It Work? | The HbA1c Level |
| Staying Active Despite the Pandemic | The Mediterranean Diet | Motivation – What Is It Really? | LDL Cholesterol |
| Turning Everyday Life into Exercise | Snacking Tips | What Are Needs? | Blood Pressure |
| Sedentary Behavior – Time to Move | Eggs: Cholesterol Bombs at Breakfast? | Acting Autonomously | Body Weight |
| It’s All About Breathing | Drinking Right | Why Motivation Isn’t Everything | - |
| Every Step Counts – Even the Smallest | Taste and Shopping | Setting Goals the Right Way | - |
| Active and Healthy in Old Age | The World of Carbohydrates | Creating Action Plans | - |
| Physical Activity During Hot Weather | Healthy Breakfast | The Power of Habits | - |
| Physical Activity and Nature | Christmas for Gourmets | Overcoming Barriers – Part 1 | - |
| Immune System and Exercise | - | Overcoming Barriers – Part 2 | - |
| Exercise and Heart Health | - | Self-Talk – A Very Special Technique | - |
| Exercise and Body Weight | - | Dealing with Negative Thoughts | - |
| Muscle Health and Well-being | - | - | - |

**Exercise videos**

Ten exercise videos were created for use at home or during peer support group meetings (see Figure S3). Each video had a specific theme and included a warm-up, a main workout, and a cool-down segment. The themes covered were:

(1) Body awareness

(2) Coordination and balance

(3) Strength training with everyday objects

(4) Cardiovascular training

A qualified sports therapist guided the sessions, providing detailed explanations for each exercise. Each video lasted approximately 30 minutes. The sessions were asynchronous, allowing participants to complete them at their own pace and according to their own schedule. No special equipment was required – participants could use common household items such as chairs, towels or water bottles. The videos ware also available for download to enable offline
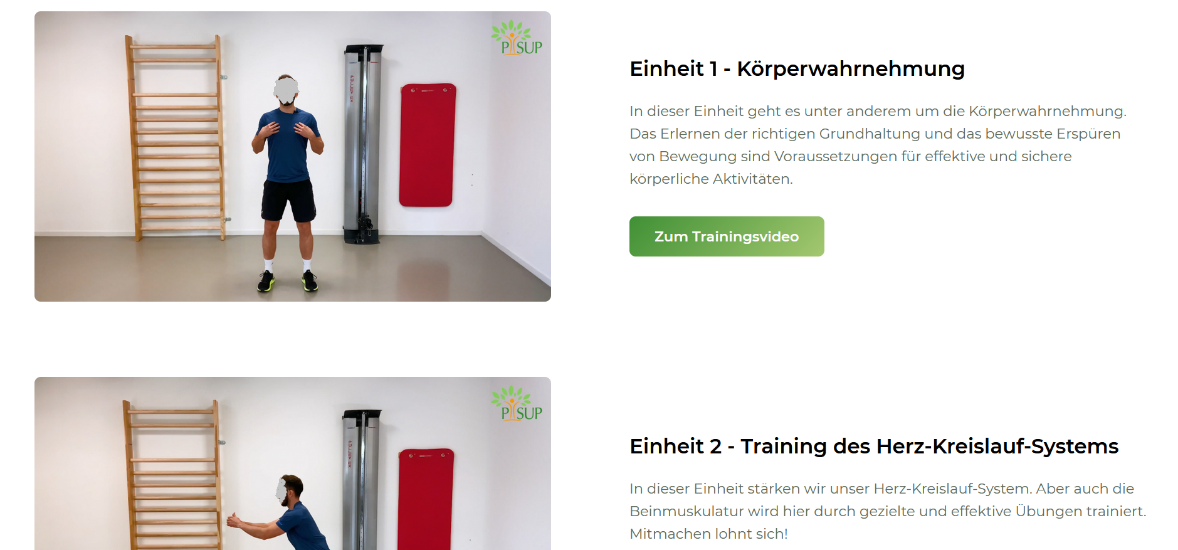
use.

Figure S3: Interface leading to the exercise videos

**Healthy recipes**


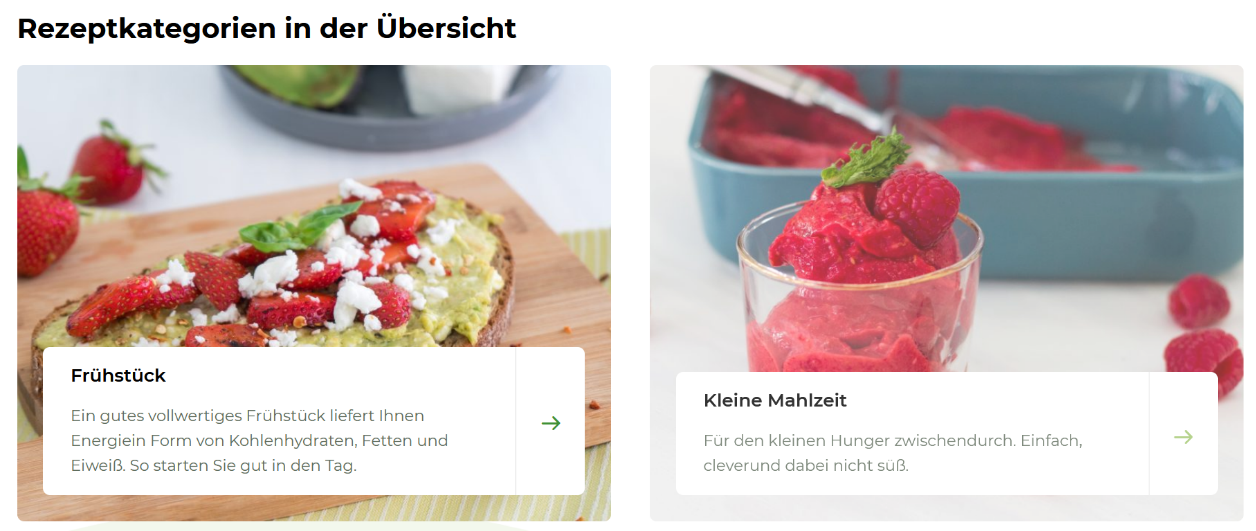
Participants were offered recipes for a balanced and healthy diet (see Figure S4). They could choose from the following categories: cold dishes, hot dishes, breakfast and snacks. All recipes were seasonally adapted and also available for download.

Figure S4: Interface leading to the recipes

**Motivational 12-week coaching**


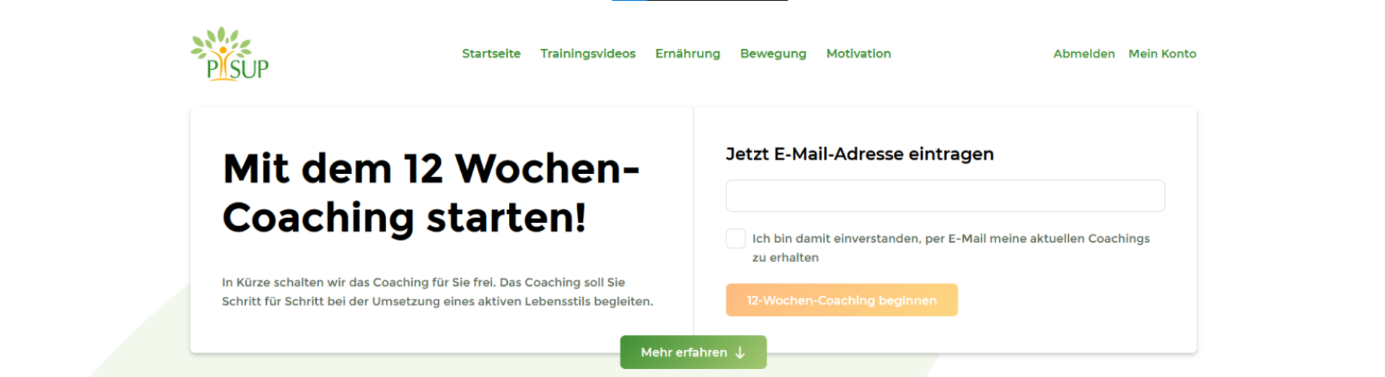
All users of the web portal had access to interactive motivational modules that incorporated various Behavior Change Techniques (BCTs) across 12 consecutive eLearning units (see Figures S5-7). This 12-week programme employed BCTs that have been shown to be effective in similar populations, including goal setting, self-monitoring and feedback [1,2]. Users received email reminders at the start of each new unit and were free to revisit completed units as often as they wished.

Figure S5: Interface leading to the 12-week coaching.


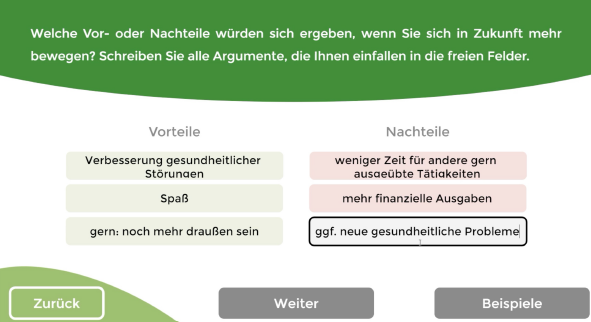


Figure S6: Module 2 – Documentation of perceived advantages and disadvantages of increasing physical activity


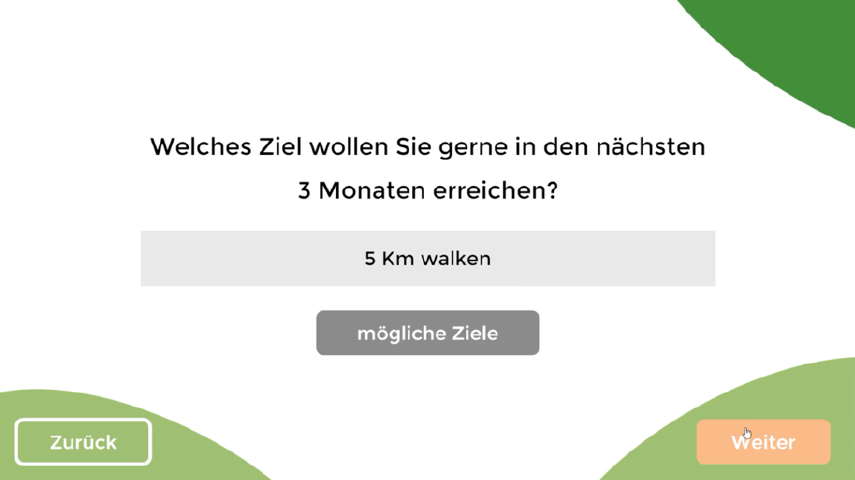
Figure S7: Module 5 – Defining an exercise goal

The 12-week coaching content was developed based on the Health Action Process Approach (HAPA) [3] and aimed to enhance motivation for increased physical activity. The programme supported participants in identifying their movement preferences, recognizing behavioural barriers and facilitating factors, engaging in self-reflection and ultimately developing a personalised physical activity plan for the future (see Table S2).

Table S2: Overview of the contents of the 12-week coaching

| **Week** | **Module title** | **Module aim** | **Module content** |
| --- | --- | --- | --- |
| **1** | Activity Check | - Understand the effects of physical (in)activity and learn about WHO recommendations  - Assess one's own physical activity  - Compare personal activity to WHO minimum requirements | - Survey of daily and exercise-related activity using the GPAQ  - Personalised feedback on current activity patterns |
| **2** | Decision Balance | - Identify perceived advantages and disadvantages of behaviour change  - Reduce one (or more) disadvantages  - Strengthen one (or more) advantages | - Collect individual perceptions of pros and cons of behaviour change  - Reduce a selected disadvantage  - Reinforce a selected advantage |
| **3** | My Exercise Motivation | - Provide knowledge on motivational models  - Self-assess personal motivation type  - Stimulate internalisation of motivation | - Information on different motivation types  - Motivation assessment using the SSK scale  - Visualisation and personalised feedback |
| **4** | Exercise Type | - Define self-concordant exercise characteristics | - Identify preferred characteristics of physical activity  - Create a personal exercise profile |
| **5** | My Exercise Goal | - Support in setting a self-concordant exercise goal | - Explaining the role of goal setting  - Support the development of a self-concordant goal  - Applying the SMART goal-setting technique |
| **6** | Imagination of the Exercise Goal | - Strenghten commitment to the previously defined goal through visualisation | - Introduction to visualisation techniques  - Guided visualization of the personal exercise goal |
| **7** | Exercise Planner | - Improve ability to develop a personal exercise or health plan  - Promote long-term integration of physical activity | - Introduction to planning techniques  - Collection of specific exercise ideas (what, where, when, how often, with whom)  - Weekly planning |
| **8** | Health Diary | - Document current health behaviours  - Raise awareness of behavioural patterns | - Introduction to self-monitoring  - Choose diary categories (exercise, diet, mood, sleep)  - Evaluate diary entries |
| **9** | Establishing a  Routine | - Integrate healthy behaviours into daily life  - Establish automatic, sustained routines | - Clarify the function of routines  - Choose a suitable routine  - Plan the daily routine |
| **10** | My Bridges | - Identify personal barriers to physical activity  - Develop individual coping strategies | - Introduction to different types of barriers  - Identification of personal barriers  - Creation of tailored solution strategies |
| **11** | Self-Reflection and Deepening | - Analyzing needs and reinforcing a behavioral change strategy | - Guided self-reflection on behavioural areas still requiring attention  - Repetition of a relevant previous module |
| **12** | My Future Self-management | - Support long-term planning of an active lifestyle | - Reflection on realistic and suitable future behaviour changes  - Develop a sustainable physical activity plan  - Complete the coaching programme |

**Download Area**


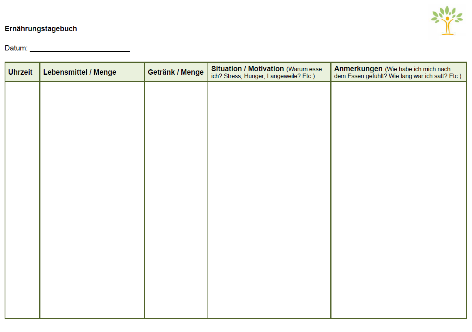
A nutrition download area was available for all participants, offering resources such as a food diary for tracking eating and drinking habits, and a weekly meal planner to support better organisation. The purpose of using a food diary was to monitor individual dietary behaviours and facilitate the adoption of new, healthier habits. Participants were encouraged to record what they ate or drank, the quantities consumed, the reasons for eating or drinking, and how they felt afterwards (see Figure S8).

Figure S8: Food diary

Printable nutritional recommendations were also provided. The recommendations covered key topics such as the plate concept, mindful eating, whole grains, fats, sources of protein, snacking, drinking, enjoyment of food and grocery planning. The plate concept offers a simple and practical approach to creating healthy, balanced meals. It is commonly used to illustrate the appropriate composition of main meals, helping individuals manage portion sizes and ensure well-balanced distribution of nutrients (see Figures S9 and 10).


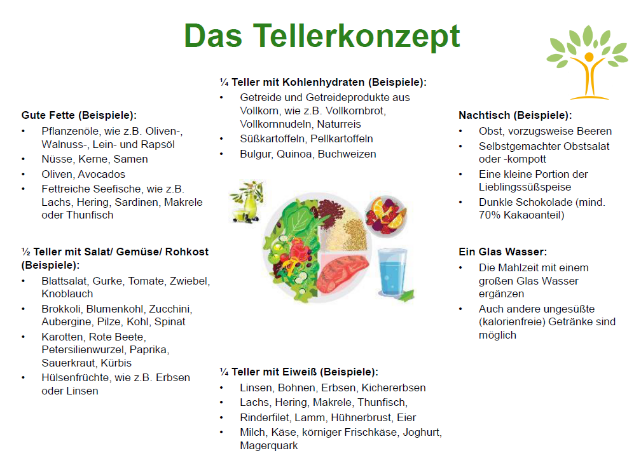

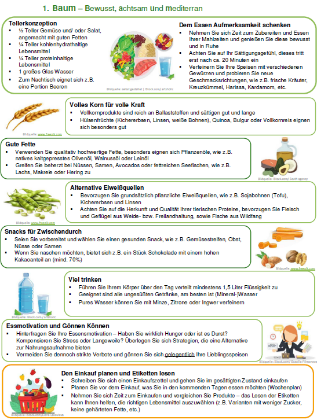
Figure S9: Nutrition Recommendations

Figure S10: Plate Concept

## **References**

1. Martín-Martín J, Roldán-Jiménez C, De-Torres I, Muro-Culebras A, Escriche-Escuder A, Gonzalez-Sanchez M, et al. Behavior Change Techniques and the Effects Associated With Digital Behavior Change Interventions in Sedentary Behavior in the Clinical Population: A Systematic Review. Front Digit Health. 2021;3:620383.

2. Zhang X, Qiao X, Peng K, Gao S, Hao Y. Digital Behavior Change Interventions to Reduce Sedentary Behavior and Promote Physical Activity in Adults with Diabetes: A Systematic Review and Meta-Analysis of Randomized Controlled Trials. Int J Behav Med. 2023 Jun 30;

3. Schwarzer R, Lippke S, Luszczynska A. Mechanisms of health behavior change in persons with chronic illness or disability: the Health Action Process Approach (HAPA). Rehabil Psychol. 2011 Aug;56(3):161–70.
