# Supplementary material for: Determinants of the Uptake and Frequency of Use of a Web Portal Digital Health Intervention in Patients With Type 2 Diabetes and/or Coronary Heart Disease: Secondary Analysis of a Randomized Controlled Trial
Source: J Med Internet Res. 2026 Mar 25;28:e80895. doi: 10.2196/80895 (PMC13016439; doi:10.2196/80895)
Supplement: Multimedia Appendix 2 [file jmir-v28-e80895-s002.docx]

**Multimedia Appendix 2.** Nutritional behavior questionnaire.

| Here are several statements about nutritional behaviour. For each statement, please indicate whether it applies to you. | | |
| --- | --- | --- |
| On average, I eat at least three handfuls of vegetables per day. | No | Yes |
| My intake of vegetables includes pulses such as lentils, beans, or peas at least twice a week. | No | Yes |
| On average, I eat at least two handfuls of fruit per day. | No | Yes |
| On average, in my household, olive oil is used at least four times per week for frying, cooking or preparing sauces or salad dressings. | No | Yes |
| On average, I drink at least 1.5 litres of water or unsweetened tea per day. | No | Yes |
| When eating cereals or cereal products (e.g., rice, flour, bread, pasta), I mainly prefer wholegrain varieties. | No | Yes |
| When shopping, I mainly prefer products with a relatively low sugar content. | No | Yes |
| If I have eaten unhealthily on one day, I compensate by eating healthily on the next day. | No | Yes |
| On most days, I refrain from eating for about four hours between main meals. | No | Yes |
