# Supplementary material for: Determinants of the Uptake and Frequency of Use of a Web Portal Digital Health Intervention in Patients With Type 2 Diabetes and/or Coronary Heart Disease: Secondary Analysis of a Randomized Controlled Trial
Source: J Med Internet Res. 2026 Mar 25;28:e80895. doi: 10.2196/80895 (PMC13016439; doi:10.2196/80895)
Supplement: Multimedia Appendix 3 [file jmir-v28-e80895-s003.doc]

**Rasch Scaling and Item Thresholds of the Patient Activation Measure (PAM-13 D)**

The 13-item PAM-13 D [1,2] consists of 13 items with a four-point response scale ranging from “does not apply” (1) to “fully applies” (4). The item scores are summed to yield a preliminary score ranging from 13 to 52. Through Rasch scaling, this score is subsequently transformed into an activation score ranging from 0.0 to 100.0 [3] (see Table S1).

| Preliminary Score | Activation |  | Preliminary Score | Activation |
| --- | --- | --- | --- | --- |
| 13 | 0,0 |  | 33 | 41,7 |
| 14 | 8,2 |  | 34 | 43,4 |
| 15 | 13,3 |  | 35 | 45,2 |
| 16 | 16,5 |  | 36 | 47,4 |
| 17 | 18,9 |  | 37 | 49,9 |
| 18 | 20,9 |  | 38 | 52,9 |
| 19 | 22,7 |  | 39 | 56,4 |
| 20 | 24,3 |  | 40 | 60,0 |
| 21 | 25,7 |  | 41 | 63,2 |
| 22 | 27,1 |  | 42 | 66,0 |
| 23 | 28,4 |  | 43 | 68,5 |
| 24 | 29,7 |  | 44 | 70,8 |
| 25 | 31,0 |  | 45 | 73,1 |
| 26 | 32,2 |  | 46 | 75,3 |
| 27 | 33,5 |  | 47 | 77,5 |
| 28 | 34,7 |  | 48 | 80,0 |
| 29 | 36,0 |  | 49 | 82,8 |
| 30 | 37,3 |  | 50 | 86,3 |
| 31 | 38,7 |  | 51 | 91,6 |
| 32 | 40,1 |  | 52 | 100,0 |

Table S1: Mapping of Preliminary Scores to Rasch-Scaled Activation Scores

**References**

1. Brenk-Franz K, Hibbard JH, Herrmann WJ, Freund T, Szecsenyi J, Djalali S, et al. Validation of the German version of the patient activation measure 13 (PAM13-D) in an international multicentre study of primary care patients. PLoS One. 2013;8(9):e74786.

2. Hibbard JH, Mahoney ER, Stockard J, Tusler M. Development and testing of a short form of the patient activation measure. Health Serv Res. 2005 Dec;40(6 Pt 1):1918–30.

3. Brenk-Franz K, Hibbard J, Tiesler F, Gensichen J. Manual zur deutschen Version des PAM 13. Institut für Allgemeinmedizin, Friedrich-Schiller-Universität, Jena; 2014.
