# Supplementary material for: Determinants of the Uptake and Frequency of Use of a Web Portal Digital Health Intervention in Patients With Type 2 Diabetes and/or Coronary Heart Disease: Secondary Analysis of a Randomized Controlled Trial
Source: J Med Internet Res. 2026 Mar 25;28:e80895. doi: 10.2196/80895 (PMC13016439; doi:10.2196/80895)
Supplement: Multimedia Appendix 4 [file jmir-v28-e80895-s004.doc]

**Correlation analysis for uptakea**

|  | | Sociodemographic variables^b^ | | | General psychological variables^c^ | | | | | | | | Topic-specific psychological variables^d^ | | | |
| --- | --- | --- | --- | --- | --- | --- | --- | --- | --- | --- | --- | --- | --- | --- | --- | --- |
|  | | Age | Gen | Edu | Ext | Agr | Ope | Con | Neu | Lon | Dep | Anx | PAc | HLi | IntPA | IntHNu |
| Sociodemographic variables^b^ | | | | | | | | | | | | | | | | |
| Age | R | 1 |  |  |  |  |  |  |  |  |  |  |  |  |  |  |
|  | *p* |  |  |  |  |  |  |  |  |  |  |  |  |  |  |  |
| Gen | R | -.002 | 1 |  |  |  |  |  |  |  |  |  |  |  |  |  |
|  | *p* | .963 |  |  |  |  |  |  |  |  |  |  |  |  |  |  |
| Edu | R | **-.181^***^** | -.083 | 1 |  |  |  |  |  |  |  |  |  |  |  |  |
|  | *p* | **<.001** | .084 |  |  |  |  |  |  |  |  |  |  |  |  |  |
| General psychological variables^c^ | | | | | | | | | | | | | | | | |
| Ext | R | **.102^*^** | .001 | .048 | 1 |  |  |  |  |  |  |  |  |  |  |  |
|  | *p* | **.028** | .976 | .324 |  |  |  |  |  |  |  |  |  |  |  |  |
| Agr | R | .006 | **.229^**^** | -.072 | .030 | 1 |  |  |  |  |  |  |  |  |  |  |
|  | *p* | .892 | **<.001** | .134 | .525 |  |  |  |  |  |  |  |  |  |  |  |
| Ope | R | -.030 | **.139^**^** | **.176^***^** | **.177^***^** | .022 | 1 |  |  |  |  |  |  |  |  |  |
|  | *p* | .525 | **.003** | **<.001** | **<.001** | .640 |  |  |  |  |  |  |  |  |  |  |
| Con | R | **.209^***^** | .050 | -.053 | **.217^***^** | **.124^*^** | .087 | 1 |  |  |  |  |  |  |  |  |
|  | *p* | **<.001** | .285 | .264 | **<.001** | **.008** | .066 |  |  |  |  |  |  |  |  |  |
| Neu | R | -.028 | **.111^*^** | -.018 | **-.278^***^** | -.085 | -.022 | **-.235^***^** | 1 |  |  |  |  |  |  |  |
|  | *p* | .555 | **.017** | .697 | **<.001** | .070 | .635 | **<.001** |  |  |  |  |  |  |  |  |
| Lon | R | -.053 | .077 | -.038 | **-.362^***^** | -.030 | -.020 | **-.241^***^** | **.303^***^** | 1 |  |  |  |  |  |  |
|  | *p* | .255 | .099 | .428 | **<.001** | .523 | .665 | **<.001** | **<.001** |  |  |  |  |  |  |  |
| Dep | R | **-.109^*^** | .062 | -.014 | **-.254^***^** | -.013 | -.008 | **-.269^***^** | **.358^***^** | **.559^***^** | 1 |  |  |  |  |  |
|  | *p* | **.019** | .187 | .775 | **<.001** | .780 | .858 | **<.001** | **<.001** | **<.001** |  |  |  |  |  |  |
| Anx | R | **-.127^**^** | **.113^*^** | -.043 | **-.243^***^** | -.049 | -.040 | **-.264^***^** | **.449^***^** | **.586^***^** | **.766^***^** | 1 |  |  |  |  |
|  | *p* | **.006** | **.015** | .362 | **<.001** | .292 | .399 | **<.001** | **<.001** | **<.001** | **<.001** |  |  |  |  |  |

**Correlation analysis for uptake^a^ (continued)**

|  | | Sociodemographic variables^b^ | | | General psychological variables^c^ | | | | | | | | Topic-specific psychological variables^d^ | | | |
| --- | --- | --- | --- | --- | --- | --- | --- | --- | --- | --- | --- | --- | --- | --- | --- | --- |
|  | | Age | Gen | Edu | Ext | Agr | Ope | Con | Neu | Lon | Dep | Anx | PAc | HLi | IntPA | IntHNu |
| Topic-specific psychological variables^d^ | | | | | | | | | | | | | | | | |
| PAc | R | .090 | **.110^*^** | .053 | **.171^***^** | **.095*** | **.153^**^** | **.272^***^** | **-.152^**^** | **-.238^***^** | **-.278^***^** | **-.232^***^** | 1 |  |  |  |
|  | *p* | .057 | **.020** | .275 | **<.001** | **.045** | **.001** | **<.001** | **.001** | **<.001** | **<.001** | **<.001** |  |  |  |  |
| HLi | R | **-.103^*^** | .031 | **.266^***^** | **.168^***^** | .017 | **.245^***^** | **.158^***^** | **-.209^***^** | **-.269^***^** | **-.286^***^** | **-.318^***^** | **.371^***^** | 1 |  |  |
|  | *p* | **.028** | .515 | **<.001** | **<.001** | .712 | **<.001** | **<.001** | **<.001** | **<.001** | **<.001** | **<.001** | **<.001** |  |  |  |
| IntPA | R | -.060 | -.035 | **.103^*^** | .089 | .014 | **.128^**^** | .062 | **-.107^*^** | -.045 | **-.117^*^** | -.089 | **.163^***^** | **.142^**^** | 1 |  |
|  | *p* | .200 | .450 | **.033** | .060 | .765 | **.006** | .191 | **.022** | .338 | **.013** | .058 | **<.001** | **.003** |  |  |
| IntHNu | R | -.074 | **-.123^**^** | **.110^*^** | .017 | .067 | **.219^***^** | .039 | -.026 | -.074 | -.089 | -.061 | **.283^***^** | **.211^***^** | **.400^***^** | 1 |
|  | *p* | .111 | **.008** | **.020** | .711 | .151 | **<.001** | .405 | .576 | .111 | .056 | .195 | **<.001** | **<.001** | **<.001** |  |

^a^Cell entries: R=correlation coefficient; *p*=p-value; N=Number of participants; Statistical significance is indicated as follows: *p < 0.05, **p < 0.01, ***p < 0.001; Correlation analyses performed on imputed data (N=462)

^b^Age=Age; Gen=Male Gender; Edu=Higher Education

^c^Ext=Extraversion; Agr=Agreeableness; Ope=Openness; Con=Consciousness; Neu=Neuroticism; Lon=Loneliness; Dep=Depression; Anx=Anxiety

^d^Pac=Patient activation; HLi=Health literacy; IntPA=Intention – Physical Activity; IntHNu=Intention – Healthy Nutrition
