# Supplementary material for: Determinants of the Uptake and Frequency of Use of a Web Portal Digital Health Intervention in Patients With Type 2 Diabetes and/or Coronary Heart Disease: Secondary Analysis of a Randomized Controlled Trial
Source: J Med Internet Res. 2026 Mar 25;28:e80895. doi: 10.2196/80895 (PMC13016439; doi:10.2196/80895)
Supplement: Multimedia Appendix 5 [file jmir-v28-e80895-s005.doc]

**Correlation analysis for frequencya**

|  | | Sociodemographic variables^b^ | | | General psychological variables^c^ | | | | | | | | Topic-specific psychological variables^d^ | | | |
| --- | --- | --- | --- | --- | --- | --- | --- | --- | --- | --- | --- | --- | --- | --- | --- | --- |
|  | | Age | Gen | Edu | Ext | Agr | Ope | Con | Neu | Lon | Dep | Anx | PAc | HLi | IntPA | IntHNu |
| Sociodemographic variables^b^ | | | | | | | | | | | | | | | | |
| Age | R | 1 |  |  |  |  |  |  |  |  |  |  |  |  |  |  |
|  | *p* |  |  |  |  |  |  |  |  |  |  |  |  |  |  |  |
| Gen | R | .138 | 1 |  |  |  |  |  |  |  |  |  |  |  |  |  |
|  | *p* | .052 |  |  |  |  |  |  |  |  |  |  |  |  |  |  |
| Edu | R | -.129 | -.098 | 1 |  |  |  |  |  |  |  |  |  |  |  |  |
|  | *p* | .073 | .177 |  |  |  |  |  |  |  |  |  |  |  |  |  |
| General psychological variables^c^ | | | | | | | | | | | | | | | | |
| Ext | R | .111 | -.054 | -.023 | 1 |  |  |  |  |  |  |  |  |  |  |  |
|  | *p* | .119 | .455 | .756 |  |  |  |  |  |  |  |  |  |  |  |  |
| Agr | R | -.051 | **.304^***^** | **-.206^**^** | .090 | 1 |  |  |  |  |  |  |  |  |  |  |
|  | *p* | .474 | **<.001** | **.004** | .210 |  |  |  |  |  |  |  |  |  |  |  |
| Ope | R | .036 | .105 | .091 | **.180^*^** | .015 | 1 |  |  |  |  |  |  |  |  |  |
|  | *p* | .623 | .145 | .208 | **.012** | .839 |  |  |  |  |  |  |  |  |  |  |
| Con | R | **.241^***^** | -.072 | -.051 | **.176^*^** | .112 | -.028 | 1 |  |  |  |  |  |  |  |  |
|  | *p* | **<.001** | .316 | .479 | **.014** | .120 | .703 |  |  |  |  |  |  |  |  |  |
| Neu | R | -.131 | **.176^*^** | .005 | **-.338^***^** | -.057 | .048 | **-.302^***^** | 1 |  |  |  |  |  |  |  |
|  | *p* | .068 | **.013** | .947 | **<.001** | .428 | .505 | **<.001** |  |  |  |  |  |  |  |  |
| Lon | R | -.042 | .127 | .023 | **-.427^***^** | -.004 | -.018 | **-.253^***^** | **.297^***^** | 1 |  |  |  |  |  |  |
|  | *p* | .554 | .074 | .749 | **<.001** | .958 | .807 | **<.001** | **<.001** |  |  |  |  |  |  |  |
| Dep | R | **-.191^**^** | .081 | .073 | **-.331^***^** | -.019 | .024 | **-.255^***^** | **.340^***^** | **.583^***^** | 1 |  |  |  |  |  |
|  | *p* | **.007** | .254 | .309 | **<.001** | .794 | .734 | **<.001** | **<.001** | **<.001** |  |  |  |  |  |  |
| Anx | R | -.139 | **.190^**^** | -.009 | **-.316^***^** | -.030 | .008 | **-.221^**^** | **.494^***^** | **.591^***^** | **.731^***^** | 1 |  |  |  |  |
|  | *p* | .051 | **.007** | .905 | **<.001** | .677 | .912 | **.002** | **<.001** | **<.001** | **<.001** |  |  |  |  |  |

**Correlation analysis for frequency^a^ (continued)**

|  | | Sociodemographic variables^b^ | | | General psychological variables^c^ | | | | | | | | Topic-specific psychological variables^d^ | | | |
| --- | --- | --- | --- | --- | --- | --- | --- | --- | --- | --- | --- | --- | --- | --- | --- | --- |
|  | | Age | Gen | Edu | Ext | Agr | Ope | Con | Neu | Lon | Dep | Anx | PAc | HLi | IntPA | IntHNu |
| Topic-specific psychological variables^d^ | | | | | | | | | | | | | | | | |
| PAc | R | .107 | -.060 | .060 | **.216^**^** | .043 | **.159^*^** | **.304^***^** | **-.173^*^** | **-.299^***^** | **-.306^***^** | **-.274^***^** | 1 |  |  |  |
|  | *p* | .133 | .400 | .409 | **.002** | .549 | **.026** | **<.001** | **.015** | **<.001** | **<.001** | **<.001** |  |  |  |  |
| HLi | R | -.030 | -.122 | **.227^**^** | **.230^**^** | -.080 | **.230^**^** | .091 | **-.163^*^** | **-.301^***^** | **-.200^**^** | **-.269^***^** | **.489^***^** | 1 |  |  |
|  | *p* | .681 | .087 | **.002** | **.001** | .264 | **.001** | .212 | **.022** | **<.001** | **.005** | **<.001** | **<.001** |  |  |  |
| IntPA | R | -.091 | .046 | **.145^*^** | .101 | -.054 | .085 | **.153^*^** | -.034 | -.026 | -.082 | -.039 | **.200^**^** | .108 | 1 |  |
|  | *p* | .199 | .518 | **.043** | .158 | .450 | .236 | **.031** | .638 | .717 | .251 | .588 | **.005** | .130 |  |  |
| IntHNu | R | -.046 | **.162^*^** | .049 | -.018 | .055 | .110 | -.005 | -.077 | -.021 | -.119 | -.029 | **.206^**^** | **.160^*^** | **.234^***^** | 1 |
|  | *p* | .515 | **.023** | .492 | .808 | .447 | .125 | .946 | .285 | .772 | .095 | .691 | **.004** | **.025** | **<.001** |  |

^a^Cell entries: R=correlation coefficient; *p*=p-value; N=Number of participants; Statistical significance is indicated as follows: *p < 0.05, **p < 0.01, ***p < 0.001; Correlation analyses performed on data completed by imputation (N=199)

^b^Age=Age; Gen=Gender; Edu=Education

^c^Ext=Extraversion; Agr=Agreeableness; Ope=Openness; Con=Consciousness; Neu=Neuroticism; Lon=Loneliness; EmDi=Emotional Distress

^d^Pac=Patient activation; HLi=Health literacy; IntPA=Intention – Physical Activity; IntHNu=Intention – Healthy Nutrition
